# Dosimetric characteristics of electron beams produced by two mobile accelerators, Novac7 and Liac, for intraoperative radiation therapy through Monte Carlo simulation

**DOI:** 10.1120/jacmp.v14i1.3678

**Published:** 2013-01-07

**Authors:** Sergio Righi, Evis Karaj, Giuseppe Felici, Fabio Di Martino

**Affiliations:** ^1^ U.O. Health Physics, Ospedali Galliera Genova Genova Italy; ^2^ Polytechnic University of Tirana Sheshi Nene Tereza Tirana Albania; ^3^ Sordina SpA Technical Division Rome Italy; ^4^ U.O. Health Physics Azienda Ospedaliero‐Universitaria Pisana Pisa Italy

**Keywords:** IORT, Monte Carlo simulation, dedicated linear accelerators

## Abstract

The Novac7 and Liac are linear accelerators (linacs) dedicated to intraoperative radiation therapy (IORT), which produce high energy, very high dose‐per‐pulse electron beams. The characteristics of the accelerators heads of the Novac7 and Liac are different compared to conventional electron accelerators. The aim of this work was to investigate the specific characteristics of the Novac7 and Liac electron beams using the Monte Carlo method. The Monte Carlo code BEAMnrc has been employed to model the head and simulate the electron beams. The Monte Carlo simulation was preliminarily validated by comparing the simulated dose distributions with those measured by means of EBT radiochromic film. Then, the energy spectra, mean energy profiles, fluence profiles, photon contamination, and angular distributions were obtained from the Monte Carlo simulation. The Spencer‐Attix water‐to‐air mass restricted collision stopping power ratios ($s_{W,\text{air}}$) were also calculated. Moreover, the modifications of the percentage depth dose in water (backscatter effect) due to the presence of an attenuator plate composed of a sandwich of a 2 mm aluminum foil and a 4 mm lead foil, commonly used for breast treatments, were evaluated. The calculated $s_{W,\text{air}}$ values are in agreement with those tabulated in the IAEA TRS‐398 dosimetric code of practice within 0.2% and 0.4% at $z_{\text{ref}}$ (reference depth in water) for the Novac7 and Liac, respectively. These differences are negligible for practical dosimetry. The attenuator plate is sufficient to completely absorb the electron beam for each energy of the Novac7 and Liac; moreover, the shape of the dose distribution in water strongly changes with the introduction of the attenuator plate. This variation depends on the energy of the beam, and it can give rise to an increase in the maximum dose in the range of 3%–9%.

PACS number: 87.56.‐v

## I. INTRODUCTION

Intraoperative radiation therapy (IORT) in its broadest sense refers to the delivery of radiation at the time of an (surgical) operation. IORT has evolved as an attempt to achieve higher effective doses of irradiation while dose‐limiting structures are surgically displaced (or adequately protected).^(^ ^1^ ^)^ The use of high‐energy electron beams is employed in the treatment of various tumor pathologies. Conventional linacs and/or dedicated accelerators may be used. In the first case, the patient is moved from the operating theater to the radiotherapy bunker in order to continue the treatment, while in the second case, special mobile linacs are employed that can execute the treatment directly in the operating theater. The use of dedicated accelerators resolves logistic and clinical problems, such as the need for transporting the anesthetized patient, thereby reducing the overall time of the procedure.

Moreover, the advent of such accelerators on the market has permitted a notable development of the methodology.^(^ ^2^ ^,^ ^3^ ^,^ ^4^ ^,^ ^5^ ^,^ ^6^ ^)^ The common characteristics of the IORT treatments are: a) a specific beam collimating system, and b) the lack of a “standard” treatment planning system based on the acquisition of the CT images, which provide the morphological information about the tissue to be treated at the moment of irradiation, as in conventional radiotherapy.

The only commercial TPS dedicated to IORT^(^ ^7^ ^)^ is instead based on CT images acquired in the preparatory phase; its use is limited, because the morphology of the tissue to be irradiated it can strongly change due to the surgical intervention.

The lack of “standard” treatment planning is due to the fact that the information related to the tissues to be irradiated would be available only at the moment of the treatment, after the surgical removal. Hence, it is only possible to evaluate the dimensions of the tissue to be irradiated — its depth and the possible presence of critical organs in the vicinity at the time of surgery — in order to choose the beam dimensions and energy and the dose delivered. The calculated dose is based on measurements performed under reference conditions.^(^ ^1^ ^)^ Therefore, since the standard dosimetry in reference conditions is the only dosimetric information available at the moment of treatment, it holds an essential role and it is fundamental that it is performed as accurately as possible.

The collimation system is obtained through special objects of PMMA (polymethyl mythacrylate) called applicators. These objects are cylindrically shaped of various lengths and dimensions, and collimate the beam directly onto the surface of the tissue to be irradiated. This type of collimation is characterized by a greater degradation of the beam energy, in comparison to electron collimators used for the conventional radiotherapy.^(^ ^8^ ^,^ ^9^ ^)^ This increases the entrance dose and modifies the stopping power ratios.^(^ ^8^ ^,^ ^9^ ^)^ The first characteristic is positive regarding the IORT treatment, because the surface of the patient is considered part of the target in these types of treatments. The second characteristic necessitates the direct calculation of the stopping power ratios if an ionization chamber is to be used, because those tabulated in the international codes are based on conventional accelerators and collimation systems.

Bjork et al.^(^ ^8^ ^,^ ^9^ ^)^ have pointed out how a conventional accelerator, equipped with a collimation system for IORT treatment, degrades the beam and modifies the stopping power ratios, but introduces an additional error of less than 1% to the dose calculation in reference conditions with a ionization chamber.

The dosimetric situation with dedicated accelerators is more critical for substantially two reasons. The first one is related to radioprotection problems regarding the use of an accelerator in an operating theater.^(^ ^10^ ^,^ ^11^ ^)^ The second reason is related to the fact that the characteristics of the beams produced vary even more from those from conventional ones not only because of the collimation system, but also because of the different design of the head. Employing the Monte Carlo code for simulating the head and the beams produced by an IORT dedicated accelerator permits one to do the following:

1) Calculate the specific stopping power ratios and improve the accuracy of the reference dosimetry with the ionization chamber.
2) Calculate the photon contamination in the beam and, in general, help evaluate the scatter and leakage radiation which are useful for the a priori radioprotection evaluation.
3) Permit the dosimetric evaluation under nonstandard irradiation conditions (inhomogeneous and nonwater‐equivalent tissues, the variation of the dosimetric distributions due to the presence of shielding for the critical organs).

In this paper, the Monte Carlo code EGSnrc^(^ ^12^ ^,^ ^13^ ^,^ ^14^ ^,^ ^15^ ^)^ is employed for simulating the head and the beams produced by two accelerators dedicated to IORT: Novac7 (New Radiant Technology SpA, Aprilia, Italy^(^ ^16^ ^)^ and Liac (Sordina SpA, Padova, Italy^(^ ^17^ ^)^.

The Novac7 produces high energy, high dose‐per‐pulse electron beams of nominal energies of 3, 5, 7, and 9 MeV, while the Liac produces energies of 4, 6, 8, and 10 MeV. Neither uses a bending magnet and the Novac7 does not use scattering foils, while the Liac has a thin scattering foil of 85 microns of brass. The beam collimation is obtained by using special cylindrical PMMA applicators of different diameters. The applicators of the Liac are smaller in length with respect to those of the Novac7; in fact, for the Novac7 the flatness of the beam is obtained only through the scattering of the electrons on the wall of the applicator. Therefore, a certain length of the applicator is necessary to obtain the flatness similar to standard radiotherapy. In the case of the Liac, this necessary length is smaller because of the presence of the scattering foil. The scattering foil is the cause for a greater degradation in the energy spectrum, a greater photon contamination, and a lower dose per pulse, compared to the Novac7. These accelerators have a dose per pulse considerably higher than that of a conventional one (up to 13 cGy/pulse for Novac7, up to 3 cGy/pulse for Liac, and around 0.1 cGy/pulse for a conventional linac). This characteristic mades it difficult, at the beginning, to use ionization chambers because of the impossibility of using methods described by the international codes regarding the evaluation of $k_{\text{sat}}$, the factor that corrects the collected charge of a ionization chamber due to the lack of complete charge collection due to ion recombination. However, various papers^(^ ^18^ ^,^ ^19^ ^,^ ^20^ ^,^ ^21^ ^)^ have described operating procedures which permit the quantification of $k_{\text{sat}}$ with good accuracy and, as a result, the possibility of using the ionization chambers as reference dosimeters. In this regard, the calculation of the specific stopping‐power ratios enables an improvement in the accuracy of measurements performed with the ionization chambers.

In this work, after simulating the heads and beams of the two accelerators and evaluating the simulations comparing the simulated dose distributions with those measured through GAFCHROMIC EBT films,^(^ ^22^ ^,^ ^23^ ^)^ the energy spectra, mean energy profiles, angular distributions, fluence profiles, photon contamination, and Spencer Attix water‐to‐air mass restricted collision stopping‐power ratios ($s_{W,\text{air}}$) were calculated. Furthermore, both the shielding properties and the influence on the dose distribution in water from special shields used in the protective breast treatment were studied. This was done for the nominal energies of 5, 7, and 9 MeV for the Novac7, and for the 6, 8 and 10 MeV for the Liac.

Beams of the Novac7 have been simulated in a previous work^(^ ^24^ ^)^ and our data basically confirm the results obtained previously and demonstrate that there is little variability between two Novac7 accelerators. Beams of this type of Liac have never been simulated before. Iaccarino et al.^(^ ^25^ ^)^ have simulated the beams produced by another version of Liac, equipped with two additional resonant cavities (to obtain a maximum nominal energy available of 12 MeV) and with a scattering foil composed by 820 μm of aluminum (for minimizing the neutron production).

For both accelerators and for all the energies, the variation in the stopping power from those tabulated in the TRS‐398 report is negligible at $z_{\text{ref}}$ ($d_{\text{ref}}$ for the TG51 report) and, as a result, it is negligible for the measurement of the absolute dose under reference conditions with an ionization chamber.

The choice of materials, which constitute the protecting shields, should be a compromise between the surgeon's requirement to have it as thin as possible, as well as the necessity to totally absorb the primary radiation and to minimize the backscatter component. The employment of Monte Carlo permits the quantification of not only the transmitting and backscatter components of a certain shield, but also the modification of the entire PDD (percentage depth dose) in water. In a previous paper,^(^ ^26^ ^)^ these evaluations using the Monte Carlo simulations have been performed on beams produced by conventional accelerators (nondedicated ones), but were also used for IORT treatment (equipped with IORT applicators for beam collimation). In this paper, the simulations have been performed for both Novac7 and Liac beams using a shield composed of a 2 mm Al foil and a 4 mm Pb foil, used in different centers which implement IORT with dedicated accelerators.

## II. MATERIALS AND METHODS

### A. Head characteristics for the two linacs

**Figure 1:**
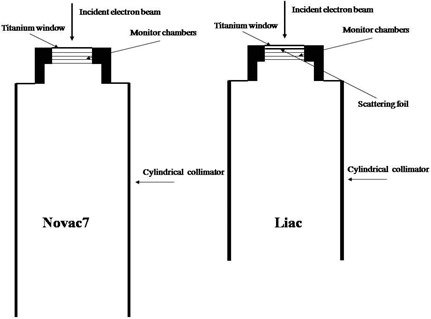
Schematic diagram of the Novac7 and Liac heads.

The head of the Novac7 (Fig. 1) is composed of the vacuum exit window (titanium), the monitor chambers (aluminum), and the applicator (PMMA). The reference applicator for dosimetry purposes and the one considered in this work has a diameter of 10 cm and a length of 100 cm. The head of the Liac (Fig. 1) is very similar to that of Novac7, except for the presence of a scattering foil of 85 μm of brass. The applicators are smaller in length with respect to those of the Novac7. The reference applicator for the Liac has a diameter of 10 cm and a length of 60 cm.

### B. Monte Carlo simulation

The 2006 release of the OMEGA/BEAM Monte Carlo system was used for these simulations. The simulation geometry includes: exit window, scattering foil in the case of the Liac, monitor unit chambers, and applicators (Fig. 1) provided by the manufacturer.

The exit foil and scattering foil (only in the case of Liac) were modeled using the SLABS component module, while for the monitor chambers and applicators, we used the CHAMBER and CIRCAPP component module, respectively. The structure that surrounds the various components was modeled using the CONESTAK component module.

The energy spectra and the geometrical shape of the electron beam at the exit window have been obtained from the manufacturers; the electron beam dynamics inside the accelerating waveguide has been simulated as a function of the accelerating electric field. The electrical field has been measured directly using a perturbative method based on Slater's perturbation method.^(^ ^27^ ^)^

In all the simulations, the energy cutoffs for particle transport were set to ECUT=0.521 MeV (kinetic energy plus rest mass) and PCUT=0.01 MeV. The EGSnrc transport parameters were taken as BCA = EXACT, electron step algorithm = PRESTA‐II with ESTEPE=0.01.

Employing the nominal energies for the Novac7 and the Liac, and taking into account the proper reference applicator, the energy spectra, the mean energy profiles, the fluences and the angular distribution of the electrons and bremsstrahlung photons at the exit of the collimator have been simulated. Moreover, the percentage of contamination photons was calculated. Each phase‐space file, extracted from the scoring plane at the collimator exit (100 cm from the exit window), was then evaluated using the BEAMDP (BEAM Data Processor) software.

The simulation of the percent depth dose (PDD) along the central axis and the dose profile at 1 cm depth was performed in water phantom by using Monte Carlo code DOSXYZnrc.^(^ ^14^ ^)^ The dimensions of voxels were chosen to be 1 mm thick and 5×5mm on the plane orthogonal to the beam axis (lateral dimensions) for the PDD. Regarding the dose profiles, the thickness is still 1 mm and the lateral dimensions 5×5mm in the flat zone of the profiles and 5×5mm in the penumbra.

The statistical uncertainty (1%) has been calculated in the regions with the lowest number of events of energy deposited (lower statistics), which is the tail for the PDD and penumbra for the dose profiles.

### C. Stopping‐power calculation

The Spencer‐Attix water‐to‐air mass restricted collision stopping‐power ratios were calculated by means of Monte Carlo code SPRRZnrc.^(^ ^15^ ^,^ ^28^ ^,^ ^29^ ^)^ The SPRRZnrc code can calculate Spencer‐Attix mass restricted collision stopping‐power ratios in each cylindrical geometry region. The code generates electron and positron fluence spectra and calculates the stopping‐power ratios, taking into account the differences in the electron and positron stopping powers explicitly.

For the calculation of collision stopping powers, the EGSnrc uses the formulae recommended by Seltzer and Berger,^(^ ^30^ ^)^ which are based on the Bethe‐Bloch theory.^(^ ^31^ ^,^ ^32^ ^,^ ^33^ ^)^ The value of the lowest energy for which secondary electrons are considered part of the electron spectra was taken to be 10 KeV, as in the major dosimetric international protocols.^(^ ^34^ ^,^ ^35^ ^,^ ^36^ ^,^ ^37^ ^)^

The stopping‐power ratios were calculated along the central axis in a region with a thickness of 2 mm specified in a column with a diameter of 4 mm inside a large water phantom, except for surface value where voxel thickness was 1 mm. The number of events (interactions) considered for the calculation was chosen to ensure a statistical uncertainty on the calculation of the stopping‐power ratios of less than 0.1% at 1 SD.

### D. PDD perturbation due to the attenuator plate

In order to evaluate the effects of the attenuation plate used to protect the healthy tissues under the target, we calculated the modified PDD along the central axes in a water phantom caused by the presence of the attenuation plate positioned perpendicular to the beam axes. The depth in water where the attenuator is positioned varies with the energy used. In clinical practice, the beam energy is chosen based on the fact that the target thickness should be completely covered at least by the 90% isodose.

Considering the experimental PDD of the Novac7 and Liac beams, we have considered depths of 16.5, 21.5, and 26.5 mm for energies 6, 8, and 10 MeV, respectively, for the Liac, and 13.5, 17.5, and 20.5 mm for 5, 7, and 9 MeV for the Novac7. The attenuator plate was composed of a sandwich of 2 mm of aluminum and 4 mm of lead. The first layer of the low‐Z material (aluminum) was introduced to reduce the backscatter component, while the second layer of high‐Z material (lead) stops the electrons.

### E. Measurements

Measurements of the PDD along the central axes and the dose profiles at 1 cm of depth were made to validate the simulations. GAFCHROMIC EBT film (International Specialty Products, Wayne, NJ) in a water phantom was used. This type of dosimeter is optimal for measurements on dedicated IORT‐beams because they are energy‐spectrum and dose‐rate independent.^(^ ^22^ ^,^ ^23^ ^)^

For calibration purposes, the films were irradiated with 12 dose values (0.15−8 Gy) using a 6 MeV electron beam produced by a conventional linac (Siemens PRIMUS, Siemens AG, Erlangen, Germany). Moreover, after being irradiated, the radiochromic films were read using an Epson Expression 1680Pro (US Epson, Long Beach, CA) with a wide spectrum (white) light. A red filter was also used to obtain the maximum absorption range (λ=636 nm). The radiochromic films were read before irradiation to know the basal optical density and 24 hours after irradiation to evaluate the change of optical density due to the absorbed dose. The curve that relates optical density and absorbed dose to water was obtained by fitting the experimental dose values. The procedure of postirradiation analysis was identical to the procedure used for calibration.

We have executed the measurements for all the beams simulated.

## III. RESULTS

**Figure 2:**
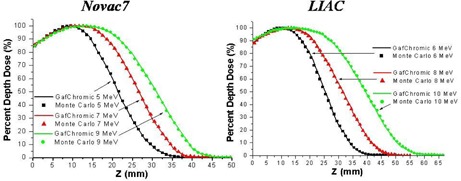
Measured (GAFCHROMIC) and simulated (Monte Carlo code) PDD in water along the beam axis.

**Figure 3:**
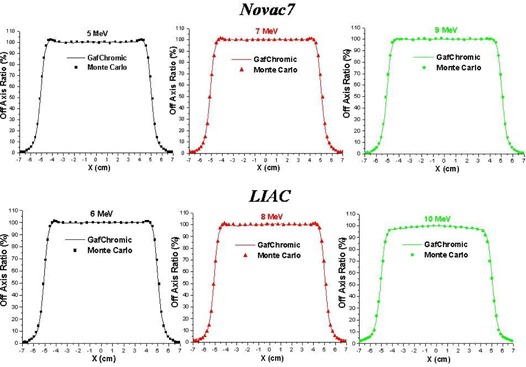
Measured (GAFCHROMIC) and simulated (Monte Carlo code) dose profiles at the depth of 1 cm in water.

The experimental measurements of the PDD along the beam axis and the dose profiles at a depth of 1 cm, performed with GAFCHROMIC films are in accordance with the Monte Carlo simulations within 2% both for the Liac and the Novac7 for all the energies considered, as we show in Fig. 2 and Fig. 3. The depths of $D_{\max}$, $R_{90}$, $R_{50}$, and $R_{p}$ for all the beams considered are reported in Table 1.

**Table 1:** PDD in water.

*Novac7*					*LIAC*				
*Energy (MeV)*	$D_{\max}(mm)$	$R_{90}(mm)$	$R_{50}(mm)$	$R_{p}(mm)$	*Energy (MeV)*	$D_{\max}(mm)$	$R_{90}(mm)$	$R_{50}(mm)$	$R_{p}(mm)$
5	10	14	21	30	6	11	17	25	34
7	12	18	27	35	8	12	21	31	43
9	13	21	31	41	10	13	27	39	53

Notes: $D_{\max}$ is the build‐up depth, $R_{90}$ and $R_{50}$ are the depth of the isodoses of 90% and 50%, respectively, and $R_{p}$ is the practical range.

**Figure 4:**
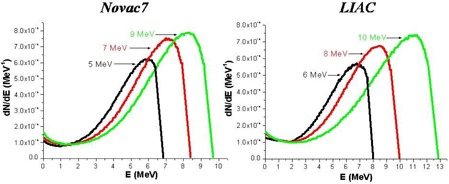
Simulated (Monte Carlo code) energetic spectrum of the electrons at the exit of collimator.

The electron energy spectra at the exit of collimator for the beams of 5, 7, and 9 MeV of Novac7 and 6, 8, and 10 MeV of Liac are shown in Fig. 4. $E_{\max}$, $E_{\text{peak}}$ (most probable), and $E_{\text{average}}$ for all the beams considered are reported in Table 2. One of the most evident characteristics in all the spectra is the presence of a ‘peak’ at the lowest energy bin. This is in principle generated by the electrons which undergo multiple scattering with the PMMA collimator wall and by the electrons interacting with the air.

**Table 2:** Energy spectrum at the exit of the collimator.

*Novac7*				*LIAC*			
*Nominal Energy (MeV)*	$E_{\mathrm{peak}}(MeV)$	$E_{\mathrm{average}}(MeV)$	$E_{\max}(MeV)$	*Nominal Energy (MeV)*	$E_{\mathrm{peak}}(MeV)$	$E_{\mathrm{average}}(MeV)$	$E_{\max}(MeV)$
5	5.9	4.7	6.8	6	6.8	5.5	8.0
7	7.1	5.9	8.4	8	8.4	6.8	10.0
9	8.2	6.8	9.7	10	11.0	9.0	12.7

Notes: Epeak is the most probable energy, Eaverage is the mean energy, and Emax is the maximum energy.

The PDDs generated from these beams demonstrate greater surface dose compared to the beams produced by the conventional accelerators with the same $R_{50}$. This is more prominent for the Liac beams in comparison to the Novac7 beams, and it is due to an increase in the number of electrons at low energy because of the presence of a scattering foil.

For the Novac7 beams, the surface dose is between 84%–86% of the maximum dose, and for the Liac beams it is 88%–91%, while the reported values in the literature for the conventional electron beams in the energy range between 6–9 MeV is around 80%.^(^ ^38^ ^)^

**Figure 5:**
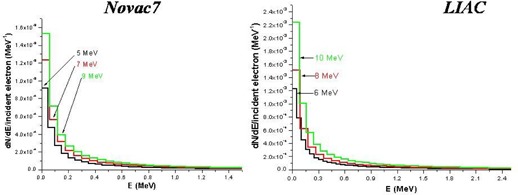
Simulated (Monte Carlo code) energetic spectrum of bremsstrahlung photons the exit of collimator.

The energy spectra of bremsstrahlung photons produced at the exit of collimator by the two linacs for all the energies employed are shown in Fig. 5. The shape of the spectrum is peaked at the lowest energies and almost without components at the high energies (> 1 MeV). Because of the reduced presence of the metallic components inside the head of the IORT‐dedicated linacs, the percentage of photon contamination (defined as percentage ratio between the photon particles versus the total particles (photons + electrons) inside the field size at the entry of the water phantom) is lower in comparison to that of conventional accelerators.^(^ ^38^ ^,^ ^39^ ^,^ ^40^ ^,^ ^41^ ^,^ ^42^ ^)^ We have calculated values of <8% and <12% for the Novac7 and the Liac accelerator photon contamination, respectively, while in the case of conventional accelerators it is in the range 20%–61%, as reported in the literature.^(^ ^38^ ^,^ ^39^ ^,^ ^40^ ^,^ ^41^ ^,^ ^42^ ^)^

**Figure 6:**
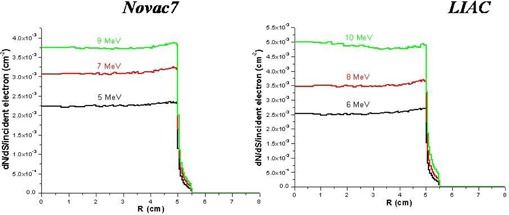
Simulated (Monte Carlo code) fluence profiles of electrons at the exit of collimator.

**Figure 7:**
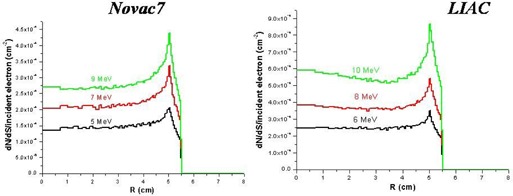
Simulated (Monte Carlo code) fluence profiles of photons at the exit of collimator.

**Figure 8:**
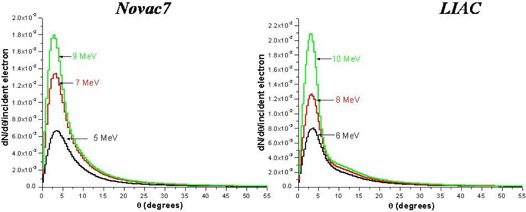
Simulated (Monte Carlo code) angular distribution of electrons at the exit of collimator.

The electron and photon fluence profiles and the electron angular distributions at the exit of the collimator for both linacs are shown in Figs. 6, 7, and 8. The electron angular distributions are characterized by an angle of maximum probability (peak), a full width at half maximum (FWHM) of the peak and a mean (average) angle; all this data are reported in Table 3.

**Table 3:** Angular distribution.

*Novac7*				*LIAC*			
*Energy (MeV)*	$\Theta_{p}(\deg rees)$	*FWHM (degrees)*	$\Theta_{m}(\deg rees)$	*Energy (MeV)*	$\Theta_{p}(\deg rees)$	*FWHM (degrees)*	$\Theta_{m}(\deg rees)$
5	3.5	6.7	9.6	6	3.5	5.6	10.1
7	3.1	5.8	9.1	8	3.1	5.0	9.5
9	2.8	5.0	8.7	10	2.7	4.2	8.6

Notes: $\Theta_{p}$ is the most probable angle, Θm is the average (mean) angle, and FWHM is the full width at half maximum of the peak of the most probable angle.

**Figure 9:**
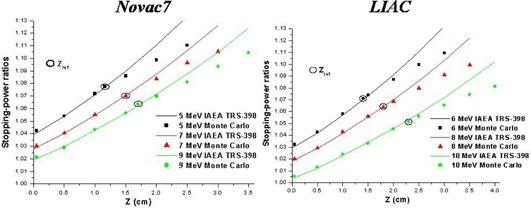
Simulated (Monte Carlo code) and tabulated (international protocol TRS‐398) water‐to‐air stopping‐power ratios.

The water‐to‐air stopping‐power ratios specific for both accelerators for all energies and those tabulated in the international protocol TRS‐398 as a function of $R_{50}$ are shown in Fig. 9.

The calculated values are higher up to $z_{\text{ref}}$, both for the Novac7 and the Liac. This reflects a major low‐energy component in the energy spectrum for the Liac and the Novac7 with respect to conventional accelerators.

At $z_{\text{ref}}$, the agreement among the tabulated values of the TRS‐398 code and those calculated are within 0.2% for the Novac7 and 0.4% for the Liac.

For depths greater than 1.5 cm for beams of the Novac7 and 2 cm for beams of the Liac, tabulated values differ from those calculated.

The difference between the tabulated stopping‐powers and the calculated ones has the following effects on the PDDs: the variation in the depth of 90% isodose (depth at which the dose is prescribed) is <0.2 mm for the two linacs and for all energies considered, and that of $R_{50}$ is <0.3 mm.

**Figure 10:**
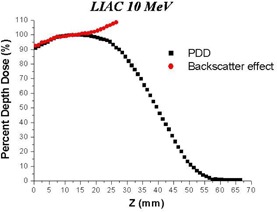
Simulated (Monte Carlo code) backscatter effect (PDD modified) caused by shield composed of a 2 mm Al foil and a 4 mm Pb foil.

Figure 10 shows the PDD modified by the presence of shielding (see Materials and Methods section above), in the case of the Liac and beam nominal energy of 10 MeV. The depth in water of the front surface of the shield is 25 mm (depth in water of the isodose level of 90%).

The backscatter component generates an increase in the maximum dose in the range 103%–109% for all the beams considered.

## IV. DISCUSSION & CONCLUSIONS

The Liac and Novac7 are electron accelerators dedicated to IORT. Their accelerator heads — and as a result, the characteristics of the beams produced — are different from those produced by conventional accelerators.

The Monte Carlo method was employed to simulate the heads of the two accelerators and to completely characterize the beams produced by them in terms of energy spectra, photonic contamination, fluence profile, angular distribution, and calculation of water‐to‐air stopping‐power ratios.

The Novac7 was simulated solely in one previous work^(^ ^24^ ^)^ and our results are in a good agreement with those reported in that study. This type of Liac has never been simulated so far, and the data obtained are described and made available to all the users. In comparison to the Novac7, the presence of a thin brass scattering foil changes the beam properties. This is reflected in higher entrance dose, in a higher photon contamination, and in a higher variation of the stopping‐power ratios with respect to those tabulated.

The water‐to‐air stopping‐power ratios calculated differ from those tabulated in the international code TRS‐398 significantly only at large depths. At the maximum dose and at $z_{\text{ref}}$ depths, such differences are negligible: 0.2% for the Novac7 and 0.4% for the Liac. This result is important. In fact, the importance and the possibility of using parallel plate ionization chambers for the dosimetry of beams produced by these linacs at very high dose‐per‐pulse has been demonstrated by different papers.^(^ ^18^ ^,^ ^19^ ^,^ ^20^ ^,^ ^21^ ^)^ These simulations show that using the values of water‐to‐air stopping‐power ratios tabulated in the international code TRS‐398 introduces a negligible additional uncertainty to the calculation of the dose at the reference depth.

We have demonstrated how the simulation of the beam may be useful for doing evaluations which are difficult to be measured. As an example, we have calculated the variation of PDD caused by the presence of a shield used in breast treatment. Various shields are used in the clinical practice. All of them have the characteristic of a sandwich, composed of material of high‐Z with the intention to completely absorb the electron beam and one composed of material with low‐Z with the intention to minimize the backscatter radiation. It is obvious that limitations are raised by the impossibility of using large thicknesses in breast surgery.

In this work, we have considered a format of 2 mm of aluminum (low‐Z material) and 4 mm of lead (high‐Z material). We have calculated the variations of PDDs for the beams of 6, 8, and 10 MeV of the Liac, and 5, 7, and 9 MeV of the Novac7. In all these cases, the transmitted component (dose to the critical organs) is less than 1% (radiological thickness greater than the practical range of the electrons for all the considered energies), and these results are in accordance with the measurements.

What is difficult to measure with accuracy and what would be useful from the clinical point of view are both the increase of dose into the target and the change of PDD due to backscatter. The maximum dose to the target can be also increased by 9%. These data may be used in clinical practice not only for adjusting the prescribed dose, but also for considering the possibility of treating targets which are deeper and cannot be covered with normal depth of 90% of isodose (the minimum isodose that should be used to cover the target).

This stresses the importance of the need to simulate the head of a dedicated accelerator. An accelerator different from a conventional one has characteristics which are less known, studied, and used in practice where there exists no TPS (treatment planning system), but in which the dosimetric knowledge are only those measured under reference conditions.

## References

[1] Beddar AS , Biggs PJ , Chang S , et al. Intraoperative radiation therapy using mobile electron linear accelerators: report of AAPM Radiation Therapy Committee Task Group No. 72. Med Phys. 2006;33(5):1476–89. 16752582 10.1118/1.2194447

[2] Gunderson LL , Willet CG , Calvo FA , Harrison LB . Intraoperative irradiation: techniques and results – 2nd edition Totowa, NJ: Humana Press; 2011.

[3] Ciocca M , Cantone MC , Veronese I , et al. Application of failure mode and effects analysis to intraoperative radiation therapy using mobile electron linear accelerators. Int J Radiat Oncol Biol Phys. 2012;82(2):e305–e311. 21708432 10.1016/j.ijrobp.2011.05.010

[4] Galimberti V , Ciocca M , Leonardi MC , et al. Is electron beam intraoperative radiotherapy (ELIOT) safe in pregnant women with early breast cancer? In vivo dosimetry to assess fetal dose. Ann Surg Oncol. 2009;16(1):100–05. 18941842 10.1245/s10434-008-0172-z

[5] Veronesi U , Orecchia R , Luini A , et al. Full dose intraoperative radiotherapy with electrons (ELIOT) during breast conserving surgery ‐ experience with 1246 cases. Available from: www.breastquestion.com/therapy/pdf/veronesi‐ecancer‐20081_opt.pdf 10.3332/eCMS.2008.65 PMC3234040 22275962

[6] Orecchia R , Luini A , Veronesi P , et al. Electron intraoperative treatment in patients with early‐stage breast cancer: data update. Expert Review Anticancer Ther. 2006;6(4):605–11. 10.1586/14737140.6.4.605 16613547

[7] GMV . Radiance. Rockville, MD: GMV; [nd]. Available from: http://www.gmv.com/en/Healthcare/radiance/

[8] Bjork P , Nilsson P , Knoos T . Dosimetry characteristics of degraded electron beams investigated by Monte Carlo calculations in a setup for intraoperative radiation therapy. Phys Med Biol. 2002;47(2):239–56. 11837615 10.1088/0031-9155/47/2/305

[9] Bjork P , Knoos T , Nilsson P . Measurements of output factors with different detector types and Monte Carlo calculations of stopping‐power ratios for degraded electron beams. Phys Med Biol. 2004;49(19):4493–506. 15552413 10.1088/0031-9155/49/19/004

[10] Krechetov AS , Goer D , Dikeman K , Daves JL , Mills MD . Shielding assessment of a mobile electron accelerator for intra‐operative radiotherapy. J Appl Clin Med Phys. 2010;11(4):3151. 21081870 10.1120/jacmp.v11i4.3151 PMC5720408

[11] Soriani A , Felici G , Fantini M , et al. Radiation protection measurements around a 12 MeV mobile dedicated IORT accelerator. Med Phys. 2010;37(3):995–1003. 20384235 10.1118/1.3298012

[12] Rogers DW , Walters B , Kawrakow I . BEAMnrc users manual. NRCC Report PIRS‐0509(A)revI. Ottawa, Canada: NRCC; 2009.

[13] Kawrakow I and Rogers DW . The EGSnrc code system: Monte Carlo simulation of electron and photon transport. NRCC Report PIRS‐701. Ottawa, Canada: NRCC; 2009.

[14] Walters B , Kawrakow I , Rogers DW . DOSXYZnrc users manual. NRCC Report PIRS‐794revB. Ottawa, Canada: NRCC; 2009.

[15] Rogers DW , Kawrakow I , Seuntjens JP , Walters BRB , Mainegra‐Hing E . NRC user codes for EGSnrc. NRCC Report PIRS‐702(revB). Ottawa, Canada: NRCC; 2010.

[16] New Radiant Technology SpA, Aprilia, Italy Available from: http://www.newrt.com/en/component/k2/itemlist/search.html?searchword=Novac7&categories=17.

[17] Sordina. Padova, Italy. Available from: http://www.sordina.com/02_products_liac.php?lan=3

[18] Di Martino F , Giannelli M , Traino AC , Lazzeri M . Ion recombination correction for very high dose‐per‐pulse high‐energy electron beams. Med Phys. 2005;32(7):2204–10. 10.1118/1.1940167 16121574

[19] Karaj E , Righi S , Di Martino F . Absolute dose measurements by means of a small cylindrical ionization chamber for very high dose per pulse high energy electron beams. Med Phys. 2007;34(3):952–58. 17441241 10.1118/1.2436979

[20] Laitano RF , Guerra AS , Pimpinella M , Caporali C , Petrucci A . Charge collection efficiency in ionization chambers exposed to electron beams with high dose per pulse. Phys Med Biol. 2006;51(24):6419–36. 17148826 10.1088/0031-9155/51/24/009

[21] Cella L , Liuzzi R , Salvatore M . The Italian affair: the employment of parallel‐plate ionization chambers for dose measurements in high dose‐per‐pulse IORT electron beams. Med Phys. 2010;37(6):2918–24. 20632603 10.1118/1.3432601

[22] ISP . GAFCHROMIC® EBT. Self‐developing film for radiotherapy dosimetry. Wayne, NJ: ISP; 2005.

[23] Butson MJ , Cheung T , Yu PK . Weak energy dependence of EBT gafchromic film dose response in the 50 kVp‐10 MVp X‐ray range. Appl Radiat Isot. 2006;64(1):60–62. 16105740 10.1016/j.apradiso.2005.07.002

[24] Pimpinella M , Mihailescu D , Guerra AS , Laitano RF . Dosimetric characteristics of electron beams produced by a mobile accelerator for IORT. Phys Med Biol. 2007;52(20):6197–214. 17921580 10.1088/0031-9155/52/20/008

[25] Iaccarino G , Strigari L , D'Andrea M , et al. Monte Carlo simulation of electron beams generated by a 12 MeV dedicated mobile IORT accelerator. Phys Med Biol. 2011;56(14):4579–96. 21725139 10.1088/0031-9155/56/14/022

[26] Martignano A , Menegotti L , Valentini A . Monte Carlo investigation of breast intraoperative radiation therapy with metal attenuator plates. Med Phys. 2007;34(12):4578–84. 18196783 10.1118/1.2805089

[27] Rosenzweig JB . Fundamentals of beam physics. Oxford, UK: Oxford University Press; 2003.

[28] Berger MJ , Selzer SM , Domen SR , Lamperti PJ . Stopping power ratios for electron dosimetry with ionization chambers. In: Biomedical Dosimetry. Vienna: IAEA; 1975 p. 589–609.

[29] Ding GX , Rogers DW , Mackie TR . Calculation of stopping‐power ratios using realistic clinical electron beams. Med Phys. 1995;22(5):489–501. 7643785 10.1118/1.597581

[30] Berger MJ and Seltzer SM . Tables of energy losses and ranges of electrons and positrons. NASA Report SP‐3012. Washington, DC: NASA; 1964.

[31] Bethe HA . Theory of passage of swift corpuscular rays through matter. Ann Physik. 1930;5:325.

[32] Bethe HA . Scattering of electrons. Z für Physik. 1932;76:293.

[33] Bloch F . Stopping power of atoms with several electrons. Z für Physik. 1933;81:363.

[34] A protocol for the determination of the absorbed dose from high energy photon and electron beams. Med Phys. 1983;10(6):741–71. 6419029 10.1118/1.595446

[35] International Atomic Energy Agency . Absorbed dose determination in photon and electron beams; an International Code of Practice. Technical Report Series No. 277. Vienna: IAEA; 1987.

[36] International Atomic Energy Agency . Absorbed dose determination in external beam radiotherapy: an international code of practice for dosimetry based on standards of absorbed dose to water. IAEA TRS‐398. Vienna: IAEA; 2004.

[37] Almond PR , Biggs PJ , Coursey BM , et al. AAPM's TG‐51 protocol for clinical reference dosimetry of high‐energy photon and electron beams. Med Phys. 1999;26(9):1847–70. 10505874 10.1118/1.598691

[38] Ding GX and Rogers DW . Energy spectra, angular spread and dose distributions of electron beams from various accelerators used in radiotherapy. PIRS‐0439. Ottawa, Canada: Institution for National Measurement Standards; 1995.

[39] Zhu TC , Bjärngard BE , Shackford H . X‐ray source and the output factor. Med Phys. 1995;22(6):793–98. 7565368 10.1118/1.597588

[40] Sheikh‐Bagheri D and Rogers DW . Sensitivity of megavoltage photon beam Monte Carlo simulations to electron beam and other parameters. Med Phys. 2002;29(3):379–90. 11930913 10.1118/1.1446109

[41] ICRU . Radiation dosimetry: electron beams with energies between 1 and 50 MeV. ICRU Report 35. Washington, DC: ICRU; 1984.

[42] Karlsson M , Nystrom H , Svensson H . Electron beam characteristics of the 50‐MeV racetrack microtron. Med Phys. 1992;19(2):307–15. 1584123 10.1118/1.596933

